# Personal Atmosphere of the Physician

**Published:** 1874-06

**Authors:** Z. Eddy


*DETROIT REVIEW OF MEDICINE AND PHARMACY—April*, 1874:

Personal Atmosphere—*By Rev. Z. Eddy.*—I cannot help adding that a physician, like a clergyman, fulfils his function as much by the immediate and unconscious influence of his personality, as by his purely professional offices. So much importance do I attach to a life-full, rich, genial personality in a physician, that I am more than half inclined to accept the theory that it is the man rather than the medicine that heals. For my own part, when I am sick I want my physician, but I am usually more benefited by his presence than by his prescriptions. Several years ago I was cured of a dangerous heart disease, and that in five minutes, by my excellent friend, Dr. Austin Flint. The cure lay in his authoritative assurance that my heart was perfectly sound. There was, however, considerable functional disturbance, which, but for my faith in the doctor, might in time have produced organic disease. While I believe in the efficacy of remedies, I have no doubt that it is usually better to take the doctor than his pills; but let him be a man pleasant and wholesome to take. I

am not a disciple of Swedenborg, but I think there is profound truth in what that extraordinary writer says about *personal atmospheres.* There are some men, and many women, whose very presence is soothing, health-giving, exhilarating, comforting. They bring with them, whenever they approach an invalid, a silent benediction and healing grace. Even sick and fretful children grow calm and bright when these ministers of health come nigh them. Such an "atmosphere" is the unconscious effluence (those who call it magnetism, odylic force, or what not, neither add to our knowledge of its nature, nor veil their own ignorance), of the full, harmonious, exuberant inner life. A man who is everywhere and always a *life-giver* and *healer*, is by divine vocation a physician. He blesses by what he *is* even more than by what he *does;* and a man becomes thus unconsciously beneficent, less by natural endowment, though that is much, than by conscientious and patient self-culture.

Another remark, of a general nature, will not, I hope, strike you as impertinent or unimportant. We of the laity have a right to require of physicians spotless purity of moral character. No man, the pastor excepted, is admitted to such intimacy in the family as the physician. He is often its most trusted friend and counselor. To him are confided the most sacred, not seldom the most painful, secrets. He has much to do with the young of both sexes, and that at the most critical age. Children grow up with the feeling that he is almost a second father; they willingly yield to his influence, and take his word for law. No man has opportunities for doing more good or more harm. Is it too much to demand that a man holding a social position so exceptionally delicate shall be clean, trustworthy, high-minded, honorable? Ought a man with tainted speech, tainted blood, tainted breath, tainted soul, to be made free of pure, refined, Christian homes? A single corrupt and unprincipled physician in any community is more to be dreaded than the cholera, the small-pox, or the plague.

